# The complete mitochondrial genome of *Semnopithecus schistaceus*

**DOI:** 10.1080/23802359.2019.1660270

**Published:** 2019-09-04

**Authors:** Ke-Ji Guo, Feng-Jun Li

**Affiliations:** a Central South Forest Inventory and Planning Institute of State Forestry Administration, Changsha, China;; b College of Life Sciences, Sichuan University, Chengdu, China

**Keywords:** Conservation genetics, Himalayan langurs, evolutionary relationships

## Abstract

*Semnopithecus schistaceus* Hodgson, 1840 belongs to subfamily Colobinae, family Cercopithecidae. This species was once mixed with *S. entellus.* The conservation status of this species is Least Concern (LC) in IUCN. In China, this species has been considered as Critically Endangered (CR) by the Red List of China’s vertebrates. In this study, the complete mitogenome of *S. schistaceus* was determined. The mitogenome is a circular molecule of 16,534 bp in length, containing 13 protein－coding genes, 2 ribosome RNA genes, 1 light strand replication origin (OL), 22 transfer RNA genes, and 1 non－coding region. We reconstructed a phylogenetic tree based on Bayesian inference for 19 primates species. The Cyt *b p*-distance is 0.029 between *S. schistaceu* and *S. entellus*. Thus, the taxonomic status of these two species remains to be further studied.

*Semnopithecus schistaceus* Hodgson, 1840 belongs to subfamily Colobinae, family Cercopithecidae, known as Himalayan langurs (Smith and Xie 2009). In 1939, Hill placed the Himalayan forms four subspecies (*hector, achilles, lanius,* and *ajax*) of *S. schistaceus*. Roonwal 1979, 1984 separated gray langurs of South Asia into a northern group (*S. schistaceus*) and a southern group (*S. entellus*) by the Tapti-Godavari rivers in central India. Groves 2001 considered *lanius* and *achilles* as junior synonyms of *S. schistaceus* but *hector* and *ajax* sufficiently distinct as to species level. Brandon-Jones 2004 listed all the Himalayan species as subspecies of *S. entellus*, but the subspecies *achilles* and *lanius* (Hill 1939) were not mentioned. Lastly in 2019, Arekar (2019) showed that Himalayan langur is a distinct species from *S. entellus* of the plains. In addition, their results did not support for splitting of the Himalayan langur into multiple subspecies. This species is distributed in NW Pakitan, N India, S China, Nepal, and W Bhutan (Allen 1938; Mittermeier et al. 2013). The conservation status of this species is Least Concern (LC) in IUCN (2019). In China, this species has been considered as Critically Endangered(CR) (Jiang et al. 2016). The species is associated with the subtropical to temperate broadleaved forest and semi-evergreen sal. They are distributed in foothills above elevations of 2000 m of the Himalayas and 3500–4000 m of Nepal (Mittermeier et al. 2013).

Up to now, no complete mitochondrial genome data of *S. schistaceus* are available in the GenBank. In this study, we sequenced the complete mitochondrial genome of *S. schistaceus* (GenBank number: MN163131) examined its phylogenetic position with other 18 mammal species.

The tissue sample was obtained from the individual which was killed by forest fire in Gyirong County, Xizang province, China, and maintained in Central South Forest Inventory and Planning Institute of State Forestry Administration, Changsha (the official accession number: GKJ2017017). Total genomic DNA was extracted from liver tissue using the DNA extraction kit (Aidlab Biotech, Beijing, China). The mitochondrial genomes of *S. entellus* (DQ355297) are used to design primers for polymerase chain reaction (PCR) and used as template for gene annotation.

The total complete mitogenome sequence of *S. schistaceus* is 16,534 bp, which is composed of 13 protein－coding genes (PCGs), 2 ribosome RNA genes, 1 light strand replication origin (OL), 22 transfer RNA genes, and 1 non－coding region. The total base composition of the *S. schistaceus* mt genome is an A + T－rich pattern of the vertebrate mitochondrial genomes. ATG is the most common start codon, ATT is used for ND2 and ND3. Most tRNAs could be folded into the canonical cloverleaf secondary structure, except tRNA－Ser(GCT). *S. schistaceus* had two non－coding regions: a 34 bp L－strand replication origin (OL) and a 1074 bp control region (D－loop).

The phylogenetic relationship for the mitochondrial genome sequences newly determined was examined with those of 17 Cercopithecidae species and two Lorisidae species. The BI analysis was performed using BEAST v1.7 (Drummond et al. 2012) and the best－fit model (GTR + G) of nucleotide evolution was selected using the Akaike information criterion (AIC) test in JModelTest 2 (Darriba et al. 2012). Phylogenetic tree resulting from the Bayesian inference (BI) analyses showed *S. schistaceu* and *S. entellus* forms a clade (pp = 1.00) as one basal position (Figure 1). However, the Cyt *b p*-distance is 0.029 between the two species. Thus, the taxonomic status of these two species remains to be further studied.

**Figure 1.**
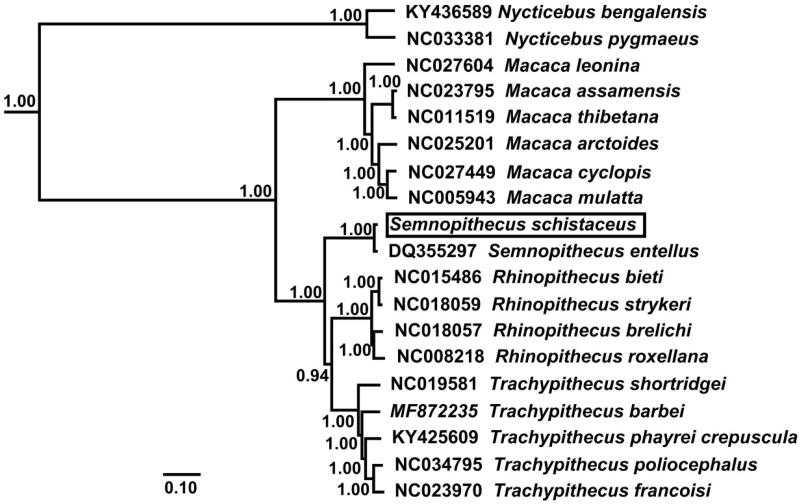
Phylogenetic tree derived from 12 protein－coding gene sequences from 19 complete mitochondrial genomes using BI analysis. Numbers by the nodes indicate Bayesian posterior probabilities.
